# Lung Volume Reduction Followed by Lung Transplantation in Emphysema—A Multicenter Matched Analysis

**DOI:** 10.3389/ti.2022.10048

**Published:** 2022-04-14

**Authors:** Alexis Slama, Laurens J. Ceulemans, Celia Hedderich, Panja M. Boehm, Jan Van Slambrouck, Stefan Schwarz, Christelle M. Vandervelde, Markus Kamler, Peter Jaksch, Dirk Van Raemdonck, Konrad Hoetzenecker, Clemens Aigner

**Affiliations:** ^1^ Department of Thoracic Surgery and Thoracic Endoscopy, University Medicine Essen, Ruhrlandklinik, Essen, Germany; ^2^ West German Center for Lung Transplantation, University Medicine Essen, Essen, Germany; ^3^ Department of Thoracic Surgery, University Hospitals Leuven, Leuven, Belgium; ^4^ Department of Chronic Diseases and Metabolism, Laboratory of Respiratory Diseases and Thoracic Surgery (BREATHE), KU Leuven, Leuven, Belgium; ^5^ Clinic of Thoracic Surgery, Medical University of Vienna, Vienna, Austria

**Keywords:** ELVR, emphysema, LVRS, lung transplantation, lung volume reduction

## Abstract

**Objective:** The impact of previous lung volume reduction surgery (LVRS) or endoscopic lung volume reduction (ELVR) on lung transplantation (LuTX) remains unclear. This study assesses the risk of previous lung volume reduction on the outcome of a later LuTX.

**Methods:** Patients suffering from emphysema who underwent bilateral LuTX were included in this multicenter analysis. Study groups were defined as: previous LVRS, previous ELVR, controls. Imbalances were corrected by coarsened exact matching for center, gender, age, diagnosis, and BMI. A comparative analysis of intraoperative characteristics, perioperative outcome and long-term survival was performed.

**Results:** 615 patients were included (LVRS = 26; ELVR = 60). Compared to controls, LVRS patients had a higher rate of postoperative ECMO (15.4 vs. 3.9%; *p* = 0.006), whereas ELVR patients suffered more often from wound infections (8.9% vs. 2.5%; *p* = 0.018). Perioperative outcome, duration of ventilation, ICU stay, and hospital stay were comparable between groups. Bacterial colonization of the airway differed significantly between both LVR groups and controls in pre- and post-LuTX cultures. Survival was not impacted (1-/3-/5-year survival for LVRS: 92.3%/85.7%/77.1%; controls: 91.3%/82.4%/76.3%; *p* = 0.58 | ELVR: 93.1%/91%/91%; controls 91.2%/81.7%/75.3%; *p* = 0.17).

**Conclusion:** Lung volume reduction does not impact short and long-time survival after bilateral LuTX. Due to differences in airway colonization after LVR, caution to prevent infectious complications is warranted.

## Graphical Abstract

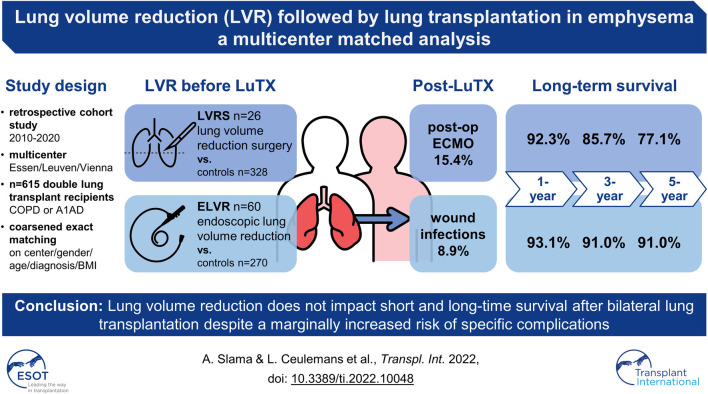

## Introduction

Patients with end-stage pulmonary emphysema have limited therapeutic options. Lung volume reduction (LVR) and lung transplantation (LuTX) have been shown to improve lung function, quality of life (QOL) and survival, despite an associated perioperative risk (1, 2). Lung volume reduction surgery (LVRS) has gained popularity in the nineties, however, remained undervalued and underused after the large NETT trial (3–5). In more recent years, endoscopic lung volume reduction (ELVR) techniques by implantation of valves or coils, thermal ablation, or instillation of chemical sealants emerged (6). However, the effect of all LVR procedures is counteracted by the progression of disease usually leading to a decay in lung function after several months to years following treatment, leaving LuTX as the last option (7). Due to the protracted clinical course of COPD in comparison to other end-stage lung diseases, the best timing for LuTX referral remains debated. Guidelines for LuTX selection recommend the use of the BODE score, which is a good predictor for pre-LuTX mortality and post-LuTX survival benefit (8).

With increased use of LVR in highly impaired “low FEV1” patients (9, 10), and the more apparent overlap of patients eligible for both LVR and LuTX, the use of LVR procedures as a “bridge to transplant” has gained acceptance (7).

Simultaneous referral of patients for both LVR and LuTX is always recommended, and the decision should be taken in an interdisciplinary emphysema board with access to all treatment options (8). Those therapies are not mutually exclusive, and most combinations have been reported, of which, in most cases, LuTX was considered the last resort when all other previous therapies failed.

In a previous review, twelve published reports on LVRS and one on ELVR preceding LuTX were identified (11). North American papers showed that LVRS before LuTX can negatively affect survival (12, 13). Other publications demonstrated only an increased perioperative risk with no impact on survival (14–16). Nevertheless, these conclusions were not supported by the most recent and largest single institution report, in which no adverse effect of a previous LVRS was observed (17).

The increased surgical risk after LVRS can potentially be attributed to a higher occurrence of adhesions and thereby a longer operation time, more bleeding complications, and a higher need for blood transfusions. Also, a higher risk of injury to the phrenic nerve during adhesiolysis has been hypothesized but was never confirmed in the available reports (11).

For ELVR prior to LuTX, available data is even more scarce, with only a single institutional analysis available (18). In 20 ELVR patients, outcome was comparable to a matched control group, although ELVR was associated with a higher occurrence of bacterial airway colonization. This observation has been recently confirmed in a larger cohort outside the scope of LuTX (19).

Given the rarity and controversy of available evidence on the impact of LVRS and ELVR on a later lung LuTX, this study aimed to further determine the short- and long-term outcomes in these patients.

## Methods

We conducted a multicenter retrospective analysis of post-LuTX outcomes in patients suffering from emphysema. Data was collected and anonymized before transmission between participating centers. This study was approved by the ethics committee of the Medical Faculty of the University of Duisburg-Essen (21-9856-BO).

### Study Population

Data was collected from three European high volume LVR and LuTX centers (Essen, Leuven, Vienna). All patients who underwent a bilateral LuTX for COPD or α-1 antitrypsin deficiency were included. The timespan for inclusion was defined individually for each center ranging from their first patient undergoing LuTX after previous LVR according to recent treatment algorithms (Essen: 2/2015; Vienna: 1/2015, Leuven: 1/2010) until August 2020. Patients with re-LuTX, unilateral LuTX or preoperative ECMO support were excluded from the analysis.

### Recipient Characteristics

Variables routinely used for listing, lung allocation score (=LAS) calculation, and organ allocation (dependent on center-specific approaches) were collected and used for analysis: age, waiting time, pack years, functional parameters, supplemental oxygen need, and pulmonary arterial pressure. LAS-data was only available for two centers. Intra-operative data included the need for size reduction (wedge or lobar LuTX), intraoperative cardiopulmonary support (ECMO, no support, CPB), duration of surgery, and cold ischemic time of both lungs independently.

### Endpoints

The primary endpoint was 1-year survival after LuTX. Secondary endpoints included duration of mechanical ventilation, time to discharge from ICU and from hospital. Surgical and medical complications were categorized and the need for postoperative ECMO was assessed. Data collection allowed for the entry of two causes of death and survival was compared to unmatched unweighted data.

### Bacterial Samples

An additional secondary endpoint of the study was to assess potential differences in airway and bronchial colonization. All positive cultures of the respiratory tract obtained while waiting for LuTX were recorded. Sputum cultures, bronchoalveolar lavage samples and swabs of the explanted recipient lung were considered. In one center, the information for every sample taken after transplantation could also be included and was used for a subgroup analysis.

### Matching

A matching algorithm was applied to reduce imbalance between groups of treated patients and controls, and to thereby improve the estimation of causal effects by statistical testing. For this purpose, “coarsened exact matching” (=CEM) was used (20). CEM has the capacity to approximate a fully blocked randomized trial unlike propensity score matching (PSM), being a less data efficient and more biased completely randomized approach (21). Coarsening was achieved by considering 5 covariates: center, gender, diagnosis, age, and BMI, the latter two with defined cut points (24, 45, 55, 65 years and 18.5, 25, 30 BMI). LAS was not used for CEM stratification as it is dependent on three of the five included variables (age, diagnosis and BMI).

### Statistics

Preprocessing of data to generate matched groups was caried out by means of R (R-Foundation for Statistical Computing, Vienna, Austria), SPSS (IBM Corp., Armonk, NY) and CEM-Extension bundle (Matthew Blackwell). All statistical analyses were conducted with SPSS v.25. Variables were assumed to be non-parametric and are reported as median and range and compared by Mann-Whitney-U tests. Nominal data were compared by means of chi‐squared test. Cases in the control groups were weighted by the CEM algorithm and frequency weight data values were rounded to the nearest integers if needed by analysis or in tables. Patient survival between the groups was compared by log‐rank (Mantel‐cox) tests on unmatched groups and unweighted matched data. Two‐sided P values <0.05 were considered statistically significant. No adjustment for multiple testing was used.

## Results

615 patients [320 (52%) female and 295 (48%) male] were included in this study. Of those, 26 (4.2%) underwent LVRS before LuTX and 60 (9.8%) had ELVR prior to transplantation. Mean age was 58 ± 5.9 years. Indications for LuTX were COPD in 572 (93%) cases and alpha-1 antitrypsin deficiency emphysema in 43 (7%) cases. In 24 (92.3%) LVRS patients, surgery took place before listing (x̃: 3.8 years; range: 0.6–32.7) whereas in two cases (7.7%), LVRS was performed while patients were on the waitlist for LuTX (2.2 and 4.4 years after listing). Median time from LVRS to LuTX was 4.0 (1.1-32.7) years. LVRS was either unilateral (15; 58%) or bilateral (11; 42%). Surgical access for LVRS was VATS in 11 (44%) cases and open surgery via thoracotomy or sternotomy in 14 (56%) cases. In one patient this information was missing. Most LVRS were performed by parenchymal stapling (*n* = 22; 85%). The remaining 4 patients underwent lobectomy (15%).

Out of 60 patients with previous ELVR, 54 (90%) had the intervention before being listed for LuTX (x̃: 2.2 years range: 0.5–6.0) and 6 (10%) on the waiting list (x̃: 2.5 years; range 0.4–6.8 after listing). Time from ELVR to LuTX was 2.7 (0.02–7.4) years. 50 (83.3%) patients had unilateral interventions and 10 (16.7%) bilateral. The procedures were: valves: 50 (83.3%), coils: 9 (15%) and a combination of both in 1 (1.7%) case. One of the patients treated with valves had hydrogel foam instilled in the contralateral apical lobe. In 20 out of 51 (39%) patients with valves, later re-intervention became necessary to either reposition or remove the valves because of unsuccessful treatment.

All 26 patients with a history of LVRS (T_LVRS_) were matched to 328 weighted controls (C_LVRS_). In patients with ELVR, 56 patents (T_ELVR_) remained in the analysis (*n* = 4 unmatched by lack of partners) and 270 weighted controls (C_ELVR_). Metrics on matching are presented in the supplementary file (Table 1).

**TABLE 1:** Demographics and patient characteristics ahead of LuTX.

		T_LVRS_ (*n* = 26)	C_LVRS_ (*n* = 328)	*p =*	T_ELVR_ (*n* = 56)	C_ELVR_ (*n* = 270)	*p =*
Gender	Female	13 (50.0%)	50.0%	1.000	29 (51.8%)	51.8%	0.988
	Male	13 (50.0%)	50.0%		27 (48.2%)	48.2%	
Diagnosis	α1-AT def.	2 (7.7%)	7.7%	0.990	3 (5.4%)	5.4%	0.963
	COPD	24 (92.3%)	92.3%		53 (94.6%)	94.6%	
Age at LuTX (y)		59 (42–70)	57 (45–74)	0.649	60 (45–72)	58 (42–74)	0.946
Waiting time (d)		180 (6–2161)	203 (2–4326)	0.506	156 (1–2932)	176 (2–3962)	0.590
BMI		21.1 (18.5–27.6)	22.5 (16.2–29.7)	0.101	22.5 (16.0–30.9)	21.6 (12.6–31.7)	0.184
Pack years		30 (0–56)	30 (0–100)	0.145	39 (0–120)	37 (0–110)	0.634
6MWT (m)		310 (20–492)	250 (0–611)	0.066	235 (0–480)	231 (0–530)	0.339
rTLC (L)		7.25 (4.03–11.1)	8 (2.89–12.1)	0.191	7.91 (4.1–12.1)	7.99 (3.26–12.6)	0.922
pTLC (L)		6.25 (4.38–7.9)	5.83 (3.65–8.2)	0.948	5.63 (3.63–7.86)	5.71 (3.98–8.67)	0.782
FEV1 (%)		21 (9.9–66)	19.6 (10–94)	0.088	19.1 (10–41)	19 (9.9–85)	0.338
PAP mmHg		32 (21–59)	32 (8–94)	0.612	34 (18–70)	31 (8–94)	0.368
LAS at listing		31.8 (29.9–35.1)	32.9 (27.8–69.7)	0.076	32.0 (29.6–38.5)	32.8 (27.8–69.7)	0.185
LAS at LuTX		32.4 (29.9–40.9)	33.4 (27.8–87.2)	***0.038***	32.6 (29.6–90.2)	33.0 (27.8–87.2)	0.223
O2 Therapy (L/min)		2 (0–6)	3 (0–15)	***0.033***	2 (0–8)	2 (0–15)	0.696
pre-LuTX hospitalization	No	25 (96.2%)	89.9%	0.306	50 (89.3%)	92.5%	0.435
	Yes	1 (3.8%)	10.1%		6 (10.7%)	7.5%	
pre-LuTX MV	No	19 (73.1%)	69.2%	0.680	41 (73.2%)	62.7%	0.297
	Noninvasive	7 (26.9%)	30.8%		15 (26.8%)	36.4%	
	ET intubation					0.9%	

Numbers are median (range) or counts (%); control columns include weighted data; significant p-values are bold; T_LVRS_, patients with previous LVRS; C_LVRS_, matched controls; T_ELVR_, patients with previous ELVR; C_ELVR_, matched controls; α1-AT def., alpha-1 antitrypsin deficiency emphysema; BMI, body mass index 6MWT, 6-min walking test; rTLC, measured total lung capacity; pTLC, predicted total lung capacity; FEV1, forced expiratory volume; PAP, pulmonary arterial pressure; LAS, lung allocation score; MV, mechanical ventilation; ET, endotracheal.

Pre-LuTX characteristics of treatment groups and matched controls are presented in Table 1. After coarsened exact matching, groups were balanced throughout demographic variables. Patients undergoing LuTX after previous LVRS had a lower median LAS at time of LuTX (32.4 vs. 33.4; *p* = 0.038) and lesser need for oxygen (2 vs. 3 L/min; *p* = 0.033); however, this was not considered of clinical relevance.

14.5% of all allocated grafts were size reduced during LuTX to match the recipient chest. Size reduction was performed either by wedge resection (*n* = 83; 13.5%) or by lobar transplantation (*n* = 6; 1.0%). 44.3% of patients had intraoperative extra corporeal support (ECS), either by ECMO (42.7%) or to a lesser extent by means of cardiopulmonary bypass (1.6%). The use of size reduction or ECS was comparable between groups. More detailed intraoperative data of both treatment groups and their weighted controls are presented in Table 2.

**TABLE 2:** Intra-operative characteristics of LuTX.

		T_LVRS_ (*n* = 26)	C_LVRS_ (*n* = 328)	*p =*	T_ELVR_ (*n* = 56)	C_ELVR_ (*n* = 270)	*p =*
Size reduction of the graft	No	19 (73.1%)	78.4%	0.532	41 (73.2%)	74.9%	0.802
	Yes	7 (26.9%)	21.6%		15 (26.8%)	25.1%	
Which size reduction	Wedge unilat.	2 (7.7%)	7.3%	0.785	2 (3.6%)	7.4%	0.512
	Wedge. bilat.	5 (19.2%)	14.2%		13 (23.2%)	15.9%	
	Lobe unilat.		0.1%			0.5%	
	Lobe bilat.					1.2%	
Intra-OP ECS	None	9 (36.0%)	33.5%	0.069	12 (21.4%)	18.1%	0.747
	CPB		5.7%		1 (1.8%)	2.4%	
	vaECMO	15 (60.0%)	60.4%		43 (76.8%)	77.9%	
	vvECMO	1 (4.0%)	0.3%			1.5%	
LuTX duration (min)		348 (137–705)	323 (150–743)	0.296	283 (150–660)	288 (137–705)	0.703
TIT 1st implanted side (min)		295 (173–577)	293 (158–812)	0.816	285 (175–542)	299 (158–698)	0.051
TIT 2nd implanted side (min)		465 (218–639)	403 (235–960)	0.101	360 (235–692)	380 (218–769)	0.180

Data expressed as median (range) or counts (%); control columns consist of weighted data; T_LVRS_, patients with previous LVRS; C_LVRS_, matched controls; T_ELVR_, patients with previous ELVR; C_ELVR_, matched controls; ECS, extra corporeal support; CPB, cardio-pulmonary bypass; vaECMO, veno-arterial extracorporeal membrane oxygenation; vvECMO, veno-venous ECMO; TIT, total ischemic time of the graft. Significant *p* values are highlited in bold italic.

Verify that all the equations and special characters are displayed correctly.In patients with previous LVRS (T_LVRS_) duration of LuTX (348 vs. 323 min; *p* = 0.296), as well as total ischemic time (TIT) of both donor lungs (465 vs. 403 min; *p* = 0.101) was statistically comparable to controls. Patients with previous ELVR did not exhibit significant differences in transplant duration and ischemic times, compared to their matched controls (283 vs. 288 min; *p* = 0.703 and 360 vs. 380 min; *p* = 0.180).

Short-term perioperative results (reoperation rates, intubation time, time on ICU and time in hospital) were excellent throughout treatment groups (T_LVRS_ and T_ELVR_). When compared to controls no clinically relevant differences were observed (Table 3). Nevertheless, in patients with previous LVRS a significantly higher rate of post-LuTX ECMO use was recorded (15.4% vs. 3.8%; *p* = 0.006).

**TABLE 3:** post-LuTX outcomes.

	T_LVRS_ (*n* = 26)	C_LVRS_ (*n* = 328)	*p =*	T_ELVR_ (*n* = 56)	C_ELVR_ (*n* = 270)	*p =*
Surgical revision	5 (19.2%)	17.7%	0.843	8 (14.3%)	16.2%	0.708
Successful weaning	24 (96.0%)	95.5%	0.894	55 (98.2%)	98.3%	0.973
Days ventilated	2 (0.5–23)	2 (0.5–79)	0.159	2 (0.5–19)	2 (0.5–79)	0.563
Post-OP ECMO	4 (15.4%)	3.8%	***0.006***	1 (1.8%)	6.4%	0.179
Post-OP ECMO (days)	7 (3–9)	4 (2–10)	0.078	2 (2–2)	5 (2–10)	0.250
Days on ICU	7 (3–213)	6 (2–152)	0.149	6 (2–107)	7 (2–152)	0.127
Days to transfer to normal ward	11 (5–213)	10 (3–118)	0.154	9 (2–107)	9 (2–118)	0.382
Days to dismissal from hospital	35 (15–105)	37 (9–152)	0.717	42 (18–109)	35 (8–152)	0.158
Death before dismissal	2 (7.7%)	8.0%	0.963	1 (1.8%)	5.8%	0.203

Control columns consist of weighted data; T_LVRS_, patients with previous LVRS; C_LVRS_, matched controls; T_ELVR_, patients with previous ELVR; C_ELVR_, matched controls; surgical revisions include all later surgical interventions on the chest and the lungs; ICU, intensive care unit. Significant *p* values are highlited in bold italic.

In T_LVRS_ complications occurred in 8 (30.8%) and in T_ELVR_ in 13 (23.2%) patients. Slight differences in the spectrum of complications were identified as LVRS patients had a higher occurrence of post-operative empyema (*n* = 2; 7.7% vs. 1.2%; *p* = 0.014). On the other hand, ELVR patients had a higher rate of wound infections (*n* = 5; 8.9% vs. 2.5%; *p* = 0.018). In deceased patients, graft failure was reported more often as cause of death in both treatment groups compared to controls (LVRS: 16.7% vs. 1.9; *p* = 0.044 | ELVR: 16.7 vs. 1.5%; *p* = 0.034). All recorded complications and causes of death are presented in Table 4.

**TABLE 4:** post-LuTX complications and causes of death.

	T_LVRS_ (n = 26; ✝: n = 6)	C_LVRS_ (n = 328; ✝: n = 70*)	*p =*	T_ELVR_ (n = 56; ✝: n = 6)	C_ELVR_ (n = 270; ✝: n = 62*)	*p =*
Complications			*0.754*			*0.444*
Pleural effusion	1 (3.8%)	10.1%		1 (1.8%)	6.8%	
Empyema/lung abscess	2 (7.7%)	1.2%	***0.014***	3 (5.4%)	3.1%	
Hemothorax	3 (11.5%)	6.7%		4 (7.1%)	6.5%	
Pneumothorax/air leak		2.7%			2.1%	
Pneumonia		2.3%		1 (1.8%)	3.0%	
Phrenic nerve injury/diaph. palsy		1.2%			2.5%	
Wound	1 (3.8%)	2.7%		5 (8.9%)	2.5%	***0.018***
Abdominal	2 (7.7%)	4.8%		3 (5.4%)	4.6%	
Arrhythmia	1 (3.8%)	2.5%		1 (1.8%)	1.1%	
ECMO related		1.1%		1 (1.8%)	1.8%	
Chest wall		1.2%			1.6%	
Sepsis		0.8%			1.8%	
PGD 3	3 (11.5%)	8.3%			2.9%	
Thrombosis. embolism		0.8%		1 (1.8%)	1.3%	
Renal failure	1 (3.8)	3.6%			1.6%	
Causes of death			*0.881*			***0.031***
Unknown	1 (16.7%)	26.9%		1 (16.7%)	14.1%	
Sepsis	1 (16.7%)	13.0%			25.0%	
Pneumonia	2 (33.3%)	18.2%		2 (33.3%)	11.1%	
MOF	1 (16.7%)	7.3%			24.1%	
GI bleeding/ischemia	1 (16.7%)	3.3%			10.6%	
Resp. insufficiency	1 (16.7%)	10.2%			25.9%	
Bleeding		2.4%			6.4%	
Graft failure	1 (16.7%)	1.9%	***0.044***	1 (16.7%)	1.5%	***0.034***
Kidney failure		1.9%			3.9%	
Malignancy		1.9%			2.2%	
Cardiac arrest/failure		3.9%		1 (16.7%)	6.9%	
CLAD		14.5%		1 (16.7%)	5.0%	
Acute/humoral rejection		6.0%			3.6%	
Pulmonary embolism					1.9%	
Euthanasia		1.1%			0.3%	
ECMO-failure		1.5%		1 (16.7%)		
Ischemic CVA		2.1%			0.2%	
Myelopathy		0.3%				

Multiple answers were allowed; complications are expressed as percentage of whole group, causes of death in relation of total deaths; T_LVRS_, patients with previous LVRS; C_LVRS_, matched controls; T_ELVR_, patients with previous ELVR; C_ELVR_, matched controls; T_LVRS_: *n* = 8 patients with complications (vs. *n* = 92*) and *n* = 6 patients died (vs. *n* = 70*); T_ELVR_: *n* = 13 with complications (vs. *n* = 56*) and *n* = 6 died (vs. *n* = 62*); PGD, primary graft dysfunction; MOF, multi organ failure; GI, gastro-intestinal; CLAD, chronic lung allograft dysfunction; CVA, cerebrovascular accident; *control columns are calculated on weighted data; in Wilcoxon–Mann–Whitney comparison of a single factor between two groups, p values >0.05 are not reported for improved readability. Significant *p* values are highlited in bold italic.

In patients with previous LVR treatment, microbiologic colonization or infection was more often detected and the spectrum of positive cultures differed significantly (LVRS: 42.3% vs. 31.1%; *p* = 0.009 | ELVR: 39.3% vs. 32.4%; *p* = 0.01) as shown in Table 5. After LuTX and immunosuppression, the rate of positive cultures increased (LVRS: 54.5% vs. 72.8%; *p* = 0.005 | ELVR: 76.9% vs. 76.8%; *p* = 0.021) and as expected certain species became more prevalent as the microbiome changed (*enterococcus* spp.*,* yeasts, mycobacteria, *aspergillus*; Table 6).

**TABLE 5:** Microbiological colonization before LuTX.

	T_LVRS_ (*n* = 26)	C_LVRS_ (*n* = 328)	*p* (%) *=*	T_ELVR_ (*n* = 56)	C_ELVR_ (*n* = 270)	*p* (%) *=*
Colonization pre-LuTX			***0.009***			***0.010***
None	15 (57.7%)	68.9		34 (60.7%)	67.6	
*Candida* sp. or YLF	8 (30.8%)	13.7		14 (25.0%)	16.8	
*Aspergillus* spp.	1 (3.8%)	7.5		4 (7.1%)	1.7	***0.022***
*Pseudomonas* spp.		6.8		1 (1.8%)	5.2	
*Staphylococcus aureus*	1 (3.8%)	5.7		4 (7.1%)	3.2	
*Klebsiella* spp.	1 (3.8%)	4.0		3 (5.4%)	2.1	
*Escherichia coli*	1 (3.8%)	1.8		2 (3.6%)	5.1	
*Serratia marcescens*	1 (3.8%)	3.1		2 (3.6%)	3.9	
*Stenotrophomonas maltophilia*	1 (3.8%)	1.4		1 (1.8%)	4.9	
*Pasteurella multocida*	1 (3.8%)			1 (1.8%)		
*Achromobacter* spp.	1 (3.8%)	0.7			0.9	
*Enterobacter cloacae complex*	1 (3.8%)	0.4	***0.037***		3.0	
*Streptococcus* spp.	1 (3.8%)	0.3	***0.027***		4.3	
Slow growing NTM		2.6		1 (1.8%)	1.9	

Species with occurrence <3% in all groups were omitted from table; control columns consist of weighted data; T_LVRS_, patients with previous LVRS; C_LVRS_, matched controls; T_ELVR_, patients with previous ELVR; C_ELVR_, matched controls; YLF, yeast like fungi; NTM, nontuberculous mycobacteria. Significant *p* values are highlited in bold italic.

**TABLE 6:** Microbiological cultures after LuTX in one center.

	T_LVRS_ (*n* = 11)	C_LVRS_ (*n* = 138)	*p* (%) *=*	T_ELVR_ (*n* = 26)	C_ELVR_ (*n* = 126)	*p* (%) *=*
Colonization post-LuTX			***0.005***			***0.021***
None	5 (45.5%)	27.2		6 (23.1%)	32.2	
*Enterococcus* spp.	5 (45.5%)	51.5		13 (50.0%)	32.3	
Slow growing NTM	3 (27.3%)	2.5	***0.000***	3 (11.5%)	5.1	
*Candida* spp. or YLF	3 (27.3%)	43.0		14 (53.8%)	41.3	
*Aspergillus species*	2 (18.2%)	14.5		7 (26.9%)	28.5	
*Staphylococcus epidermidis*	1 (9.1%)			2 (7.7%)		
*Enterobacter cloacae complex*		2.5		3 (11.5%)	3.3	
*Stenotrophomonas maltophilia*		9.3		2 (7.7%)	11.1	
*Escherichia coli*		4.2		1 (3.8%)	2.6	
*Klebsiella* spp.		2.5		1 (3.8%)	9.8	
*Achromobacter* spp.		3.4			2.6	
*Staphylococcus aureus*		3.2			7.8	
*Citrobacter freundii*		0.8			3.3	
Rapid growing NTM		0.8			3.3	
*Pseudomonas* spp.		10.3			18.2	

Percentages <3% in all groups were omitted; control columns consist of weighted data; T_LVRS_, patients with previous LVRS; C_LVRS_, matched controls; T_ELVR_, patients with previous ELVR; C_ELVR_, matched controls; NTM, nontuberculous mycobacteria; YLF, yeast like fungi. Significant *p* values are highlited in bold italic.

**FIGURE 1:**
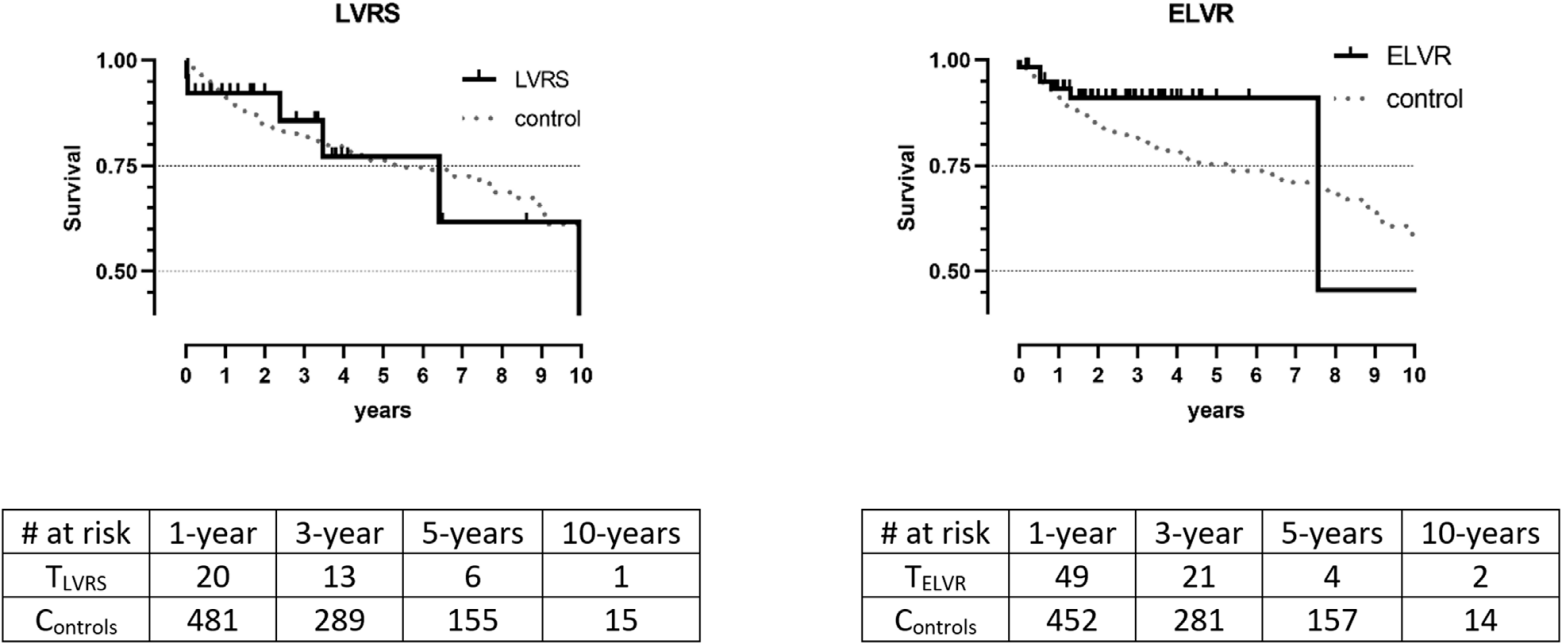
Kaplan Meier survival between treatment groups and controls. Data is unmatched and unweighted; *T_LVRS_*: patients with previous *L_VRS_*; *C_LVRS_*: matched controls; *T_ELVR_*: patients with previous *E_LVR_*. *C_ELVR_*: matched controls; no significant differences in Log-Rank tests.

Short and long-term survival after LuTX was excellent across groups and controls and consistently comparable with the control groups (1-/5-year: LVRS: 92.3%/77.1%/*p* = 0.583|ELVR: 98.3%/91.0%/*p* = 0.174). Median survival after LuTX was: LVRS: 9.95 years and ELVR: 7.56 years. Median survival for controls was not calculated as more than 50% of those patients were still alive at time of analysis (Figure 1).

## Discussion

This multi-center matched retrospective cohort study, assessed post-operative outcomes of emphysema patients, who underwent surgical or endoscopic LVR prior to LuTX. After matching, baseline indicators and intraoperative modalities were comparable between groups and controls. The analysis showed that both previous LVRS and ELVR were associated with a different spectrum of bacterial colonization prior to LuTX which must be considered to prevent infectious complications. LVR did not impact short- and long-term survival, which was equally good in all groups.

Interestingly we observed that patients with previous LVRS had a higher rate of post-operative ECMO need after LuTX. This might be explained by a longer and more difficult preparation due to pleural adhesions. Hence, potentially leading to longer surgery durations/ischemic times and an increased need for blood transfusions, all known risk factors for early mortality and PGD (22–24). Three cases of PGD 3 within 72 h occurred in the LVRS group. In previous publications, an association of previous LVRS with a higher pulmonary arterial pressure and an increased risk of phrenic nerve palsy was postulated (13, 15). These assumptions are not corroborated by our data as median PAP was 32 mmHg and not a single case of phrenic nerve injury was observed in the LVRS group. Additionally, it is noteworthy that in this cohort, the outcome in LVRS patients was statistically comparable to the outcome in patients with previous non-LVRS intrathoracic surgery.

To the best of our knowledge, this study is the largest published series about ELVR prior to LuTX. Those patients had equally good perioperative outcomes as controls although a higher occurrence of wound infections (8.9% vs. 2.5%; *p* = 0.018) was observed. Out of those five patients, four had positive sputum cultures (C*andida/Aspergillus/Klebsiella*) before LuTX. After LuTX, these patients suffered also from empyema *n* = 3 and pneumonia *n* = 1. Although the association between ELVR and post-LuTX wound infection is not fully understood, we hypothesize that a different spectrum of pre-transplant colonization and an increased exposure to antibiotics might make them more susceptible to hospital acquired infections and multi-drug resistant bacteria.

This study is the first to assess extensively airway colonization of LuTX recipients who underwent previous LVRS or ELVR. It showed that LVR was associated with a distinct airway microbiome both before and after LuTX. In comparison to other LuTX-indications COPD has a lower risk of bacterial infections (25). It was unexpected that the number of colonized patients were as high even before LuTX (LVRS: 42.3%; ELVR: 39.3%). Unfortunately, data on colonization after LuTX was only available for one of the three participating centers. In this subgroup, colonization rates of 54% in LVRS patients and 76.9% in ELVR patients were seen after LuTX.

In LVRS patients a higher rate of slow growing mycobacteria was observed after LuTX in comparison to the control group (27.3% vs. 2.5%; *p* < 0.001), an observation which cannot be readily explained. On the other hand, ELVR has been previously associated with pathological colonization as implanted valves and coils impede mucus clearance (26). Although a predominance of *stenotrophomonas maltophilia* (40%) after ELVR and LuTX has been described (18), this relationship could not be confirmed by our data in which only 7.7% patients presented with *s.maltophilia* after LuTX.

The strength of this study and its conclusion is given by its design. Three high volume centers experienced in both LVR and LuTX provided data on all patients recently transplanted for emphysema at their institution. To correct for selection bias and differences in patient characteristics, LVRS and ELVR patients were matched to weighted control groups. This led to highly balanced groups.

The observations of this study are mostly in line with a recent single-center analysis comparing 52 LVRS+LuTX patients to 65 unmatched controls (17). However, our findings differed markedly from those of a recent UNOS-database analysis (12) which included 106 LVRS+LuTX patients (from 37 LuTX centers), propensity matched to 106 controls without previous intrathoracic surgery (from 67 LuTX centers). This UNOS analysis identified a significantly increased risk of death (HR: 1.72; CI: 1.13-2.6; *p* = 0.01) after LVRS+LuTX, which was surprisingly not associated with the total number of LuTX (HR: 0.99) or the total number of LVRS+LuTX (HR: 0.99) of individual centers. Furthermore, the observed median survival was significantly worse in LVRS patients in comparison to matched controls (3.4 vs. 6.5; *p* = 0.038).

The present study has several limitations. First, no donor specific characteristics apart from ischemic times of the donor lungs were taken into consideration. Secondly, there is substantial heterogeneity in how LVRS was performed. LVRS nowadays is routinely performed by a bilateral video-thoracoscopic approach and we can hypothesize that such a minimally invasive approach would have a lesser associated risk in a latter LuTX. In this cohort, sternotomy, thoracotomy, VATS, pleurectomy, pleurodesis and pleural tenting were in use and inevitably lead to pleural adhesions, albeit to different extent. By the sample size of 26 LVRS patients, this cannot be sufficiently considered. A similar limitation applies to different ELVR approaches (valves, coils, foam, vapor) having a different risk profile (pneumonia, exacerbation and pneumothorax) (27) and suggesting that their impact on a later LuTX may differ. A possible observer bias must be addressed with regards to the microbiological cultures. Although recipient bronchi were all sampled during LuTX, patients who underwent previous ELVR had supposedly more bronchoscopies and therefore more samples taken before transplant.

Most patients had LVR before being listed for transplantation (*n* = 78). “Bridging to LuTX” only took place in 8 patients who were already on the waiting list. Waiting time for LuTX was comparable throughout groups as presented in Table 1 (LVRS 180 days vs. 203 days in controls; ELVR 156 vs. 176 days in controls).

Additionally, critically ill patients who underwent LVR and were not later referred to LuTX (because of improvement, complications, or clinical misjudgment) were not considered. Hence, this study cannot predict the impact of LVR as an alternative to LuTX. It did not account for functional improvements while waiting, nor for changes in LAS scores and impact on waiting times. The crucial question about QOL, functional/survival benefits, and timing of LVR before LuTX cannot be answered and the authors recommend further prospective investigation to answer it.

## Conclusion

This study clearly demonstrates that patients who underwent previous surgical or endoscopic LVR can safely be considered for later LuTX. Although a marginally increased risk of specific complications and differences in airway colonization after LuTX were observed, short- and long-term survival was very good.

## Data Availability

The raw data supporting the conclusion of this article will be made available by the authors, without undue reservation.

## Abbreviations

BMI, body mass index; CEM, coarsened exact matching; CLAD, chronic lung allograft dysfunction; COPD, chornic obstructive pulmonary desease; CPB, cardio pulmonary bypass; CVA, cerebro-vascular accident; ECMO, extra-corporeal membrane oxygenation; ECS, extra corporeal support; ELVR, endoscopic lung volume reduction; ICU, intensive care unit; LAS, lung allocation score; LuTX, lung transplantation; LVR, lung volume reduction; LVRS, lung volume reduction surgery; MOF, multi-organ failure; 6MWT, six minutes walking test; PGD, primary graft dysfunction; QOL, quality of life; VATS, video assisted thoracic surgery.

## References

[1] van Agteren JE Carson KV Tiong LU Smith BJ. Lung Volume Reduction Surgery for Diffuse Emphysema. Cochrane Database Syst Rev (2016) 10:CD001001. 10.1002/14651858.CD001001.pub3 27739074 PMC6461146

[2] Singer L Singer J. Quality of Life in Lung Transplantation. Semin Respir Crit Care Med (2013) 34:421–30. 10.1055/s-0033-1348470 23821515 PMC4238962

[3] Criner GJ Cordova F Sternberg AL Martinez FJ. The National Emphysema Treatment Trial (NETT). Am J Respir Crit Care Med (2011) 184:881–93. 10.1164/rccm.201103-0455ci 21719757 PMC3208657

[4] Naunheim KS Wood DE Mohsenifar Z Sternberg AL Criner GJ DeCamp MM Long-term Follow-Up of Patients Receiving Lung-Volume-Reduction Surgery versus Medical Therapy for Severe Emphysema by the National Emphysema Treatment Trial Research Group. Ann Thorac Surg (2006) 82:431–43. 10.1016/j.athoracsur.2006.05.069 16888872

[5] Fishman A Martinez F Naunheim K Piantadosi S Wise R Ries A A Randomized Trial Comparing Lung-Volume-Reduction Surgery with Medical Therapy for Severe Emphysema. N Engl J Med (2003) 348:2059–73. 10.1056/NEJMoa030287 12759479

[6] Gompelmann D Eberhardt R Herth FJF. Endoscopic Lung Volume Reduction. A European Perspective. Ann ATS (2013) 10:657–66. 10.1513/annalsats.201301-003fr 24364770

[7] Darwiche K Aigner C. Clinical Management of Lung Volume Reduction in End Stage Emphysema Patients. J Thorac Dis (2018) 10:S2732–S2737. 10.21037/jtd.2018.02.69 30210825 PMC6129813

[8] Weill D Benden C Corris PA Dark JH Davis RD Keshavjee S A Consensus Document for the Selection of Lung Transplant Candidates: 2014-An Update from the Pulmonary Transplantation Council of the International Society for Heart and Lung Transplantation. J Heart Lung Transplant (2015) 34:1–15. 10.1016/j.healun.2014.06.014 25085497

[9] Darwiche K Karpf-Wissel R Eisenmann S Aigner C Welter S Zarogoulidis P Bronchoscopic Lung Volume Reduction with Endobronchial Valves in Low-FEV1 Patients. Respiration (2016) 92:414–9. 10.1159/000452629 27838695

[10] Trudzinski FC Höink AJ Leppert D Fähndrich S Wilkens H Graeter TP Endoscopic Lung Volume Reduction Using Endobronchial Valves in Patients with Severe Emphysema and Very Low FEV1. Respiration (2016) 92:258–65. 10.1159/000448761 27603781

[11] Slama A Taube C Kamler M Aigner C. Lung Volume Reduction Followed by Lung Transplantation-Considerations on Selection Criteria and Outcome. J Thorac Dis (2018) 10:S3366–S3375. 10.21037/jtd.2018.06.164 30450243 PMC6204338

[12] Krishnan A Chidi A Merlo CA Shah PD Ha J Higgins RSD Lung Volume Reduction Surgery Prior to Lung Transplantation: A Propensity-Matched Analysis. Ann Thorac Surg (2021) 113(2):491–7. 10.1016/j.athoracsur.2021.02.009 33609545

[13] Backhus L Sargent J Cheng A Zeliadt S Wood D Mulligan M. Outcomes in Lung Transplantation after Previous Lung Volume Reduction Surgery in a Contemporary Cohort. J Thorac Cardiovasc Surg (2014) 147:1678–83. 10.1016/j.jtcvs.2014.01.045 24589202

[14] Burns KEA Keenan RJ Grgurich WF Manzetti JD Zenati MA. Outcomes of Lung Volume Reduction Surgery Followed by Lung Transplantation: a Matched Cohort Study. Ann Thorac Surg (2002) 73:1587–93. 10.1016/s0003-4975(02)03499-9 12022555

[15] Shigemura N Gilbert S Bhama JK Crespo MM Zaldonis D Pilewski JM Lung Transplantation after Lung Volume Reduction Surgery. Transplantation (2013) 96:421–5. 10.1097/tp.0b013e31829853ac 23736352

[16] Wisser W Deviatko E Simon-Kupilik N Senbaklavaci Ö Huber ER Wolner E Lung Transplantation Following Lung Volume Reduction Surgery. J Heart Lung Transplant (2000) 19:480–7. 10.1016/s1053-2498(00)00085-1 10808156

[17] Inci I Iskender I Ehrsam J Caviezel C Hillinger S Opitz I Previous Lung Volume Reduction Surgery Does Not Negatively Affect Survival after Lung Transplantation†. Eur J Cardiothorac Surg (2018) 53:596–602. 10.1093/ejcts/ezx318 28957998

[18] Fuehner T Clajus C Fuge J Jonigk D Welte T Haverich A Lung Transplantation after Endoscopic Lung Volume Reduction. Respiration (2015) 90:243–50. 10.1159/000434685 26138023

[19] Sarmand N Gompelmann D Kontogianni K Polke M Herth FJ Eberhardt R. New Bacterial Growth in Bronchial Secretions after Bronchoscopic Valve Implantation. Int J Chron Obstruct Pulmon Dis (2018) 13:565–70. 10.2147/copd.s148196 29445273 PMC5810521

[20] Iacus SM King G Porro G. Cem: Software for Coarsened Exact Matching. J Stat Softw (2009) 30:1–27. 10.18637/jss.v030.i09 21666874

[21] King G Nielsen R. Why Propensity Scores Should Not Be Used for Matching. Polit Anal (2019) 27:435–54. 10.1017/pan.2019.11

[22] Altun GT Arslantaş MK Cinel İ. Primary Graft Dysfunction after Lung Transplantation. Turk J Anaesthesiol Reanim (2015) 43:418–23. 10.5152/TJAR.2015.16443 27366539 PMC4894186

[23] Borders CF Suzuki Y Lasky J Schaufler C Mallem D Lee J Massive Donor Transfusion Potentially Increases Recipient Mortality after Lung Transplantation. J Thorac Cardiovasc Surg (2017) 153:1197–203. 10.1016/j.jtcvs.2016.12.006 28073574 PMC5392422

[24] Weber D Cottini SR Locher P Wenger U Stehberger PA Fasshauer M Association of Intraoperative Transfusion of Blood Products with Mortality in Lung Transplant Recipients. Perioper Med (2013) 2:20. 10.1186/2047-0525-2-20 PMC3964322 24472535

[25] Paglicci L Borgo V Lanzarone N Fabbiani M Cassol C Cusi MG Incidence and Risk Factors for Respiratory Tract Bacterial Colonization and Infection in Lung Transplant Recipients. Eur J Clin Microbiol Infect Dis (2021) 40(6):1271–82. 10.1007/s10096-021-04153-1 33479881 PMC8139905

[26] Fruchter O Rosengarten D Goldberg E Ben-Zvi H Tor R Kramer MR. Airway Bacterial Colonization and Serum C-Reactive Protein Are Associated with Chronic Obstructive Pulmonary Disease Exacerbation Following Bronchoscopic Lung Volume Reduction. Clin Respir J (2016) 10:239–45. 10.1111/crj.12211 25196428

[27] Ramaswamy AA Puchalski J. Bronchoscopic Lung Volume Reduction: Recent Updates. J Thorac Dis (2018) 10:2519–27. 10.21037/jtd.2018.02.72 29850160 PMC5949452

