# Supplementary material for: Lung Volume Reduction Followed by Lung Transplantation in Emphysema—A Multicenter Matched Analysis
Source: Transpl Int. 2022 Apr 14;35:10048. doi: 10.3389/ti.2022.10048 (PMC9047703; doi:10.3389/ti.2022.10048)
Supplement: Supplementary file 1 [file Table1.docx]

|  |  | LVRS | ELVR |
| --- | --- | --- | --- |
| initial imbalance measure (L1) |  | 0.968 | 0.919 |
| imbalance after matching (L1) | center | 0 | 0 |
|  | gender | 0 | 0 |
|  | diagnosis | 0 | 0 |
|  | age | 0.102 | 0.024 |
|  | BMI | 0.198 | 0.111 |
| Treatment group (n) |  | 26 | 56 |
| matched controls (n) |  | 328 | 270 |
| unmatched (n) |  | 261 | 289 |

Table 1: metrics on imbalance prior and after matching; matching was performed by „coarsened exact matching“ (=CEM) with 5 covariates (ceter, diagnosis, age, gender, BMI)
